# Evaluation of mainstreaming youth-friendly health in private clinics in Malawi

**DOI:** 10.1186/s12913-020-4937-9

**Published:** 2020-02-03

**Authors:** Janine Barden-O’Fallon, Shara Evans, Chrissie Thakwalakwa, Witness Alfonso, Ashley Jackson

**Affiliations:** 1 MEASURE Evaluation, Carolina Population Center, University of North Carolina at Chapel Hill, Chapel Hill, USA; 2 Department of Maternal & Child Health, Gillings School of Global Public Health, University of North Carolina at Chapel Hill, Chapel Hill, USA; 3 Centre for Social Research, Chancellor College, Zomba, Malawi; 4 Institute of Public Opinion and Research, Zomba, Malawi; 5 Population Services International (PSI), Washington, D.C, USA

**Keywords:** Youth-friendly health services, Family planning, Youth, Adolescents, Malawi, Evaluation, Qualitative data

## Abstract

**Background:**

High fertility rates and low modern contraceptive use put African youth and adolescents at high risk for health complications, including maternal mortality. Mainstreaming youth-friendly health services (YFHS) into existing services is one approach to improve access to reproductive health services for youth and adolescents. The objective of the evaluation was to assess the effects of a Population Services International (PSI)-sponsored YFHS training package on voluntary uptake of family planning among youth and perceptions of service quality by youth and trained healthcare providers in Malawi.

**Methods:**

In 2018, a mixed-methods convergent parallel design was used to assess relevant monitoring and evaluation documents and service statistics from PSI Malawi and qualitative data on perceptions of service quality from Malawian youth and healthcare providers. The data were assessed through separate descriptive and thematic analysis and integrated to generate conclusions.

**Results:**

Results show that the number of family planning clients ages 15–24 increased from 72 to 2278 per quarter during the implementation of the YFHS training packages, however, positive trends in client numbers were not sustained after youth outreach activities ended. Focus group discussions with 70 youth and adolescents indicated that clinics were perceived as providing high-quality services to youth. The main barriers to accessing the services were cost and embarrassment. Interviews with ten healthcare providers indicated that many made efforts to improve clinic accessibility and understood the barrier of cost and importance of outreach to youth and the broader community.

**Conclusions:**

The findings support research showing positive effects of mainstreaming YFHS when training for healthcare staff is combined with additional YFHS programming components. Furthermore, the findings provide evidence that provider training alone, though beneficial to perceived service quality, is not sufficient to sustain increases in the number of adolescent and youth family planning clients.

## Background

Adolescent fertility is higher in Africa than in any other part of the world, at 108 births per 1000 women ages 15–19 [1]. Compared to adults, adolescents who give birth are at higher risk for death, health complications, and long-term economic and social consequences [2, 3]. Yet, adolescents (ages 15–19) and young people (ages 15–24) face unique individual, interpersonal, institutional, and community-level barriers to exercising their rights to make and act on decisions about their reproductive health (RH) and to access voluntary modern contraception [4–7]. These barriers include provider biases about serving youth, insufficient supply of RH services for youth clients, and lack of national policies and guidelines, as well as barriers due to internalized stigma and reluctance to seek care, among others [5–7]. Efforts to improve access to RH services for female and male adolescents and youth have frequently focused on providing stand-alone youth health clinics that offer contraceptive and RH services in separate buildings, rooms, or spaces from older clients. These have shown positive effects in reducing adolescent pregnancies and improving access to modern contraception [4, 8–12].

However, stand-alone clinics are often more expensive and difficult to scale up because they require additional infrastructure and staffing [4, 5, 13]. The costs of providing parallel services for youth and adults is particularly challenging in resource-constrained settings [4, 7, 11, 14–16]. An alternative solution to increase access to voluntary family planning (FP) and modern contraception is through mainstreaming youth-friendly health services (YFHS) into already existing services, thereby minimizing costs and creating more sustainable voluntary FP services [11, 17]. The evidence for mainstreaming youth-friendly elements into existing FP services is limited, especially for large-scale and/or sustained programming, but shows positive effects on increasing adolescent contraceptive use [8, 12, 17–19]. The WHO Quality of Care Framework standards for YFHS are services that are equitable, accessible, acceptable, appropriate, effective and gender equitable [20]. Provision of services that have these attributes to youth and adolescents has been shown to have positive effects on uptake of modern contraception and reduced pregnancy and abortion among adolescents and youth [21]. Evidence also shows that lack of youth-friendly training and youth-friendliness among providers is a substantial barrier to adolescents’ and young people’s use of contraceptive and FP services [22–25].

### Program description

Since 2010, the Support for International Family Planning Organizations (SIFPO) and SIFPO2 projects at Population Services International (PSI) have focused on increasing access to and voluntary use of high-quality, affordable FP. Capacity building for implementation of YFHS has been an integral part of the work to expand access to voluntary FP and contraceptive choice. During this time, PSI provided its country network members with a youth programming guide for healthcare providers, service administrators, program implementers, researchers, and planners [26]. The guide presented an evidence-based overview of the need for YFHS and key recommendations for developing, implementing, and evaluating YFHS [26]. The guide included tools and checklists for clinics to evaluate YFHS delivery at their site, interactions between patients and providers, and overall patient satisfaction [26]. PSI also created a YFHS certification tool that helped to ensure that these practices were put into place and were followed within each individual clinic [26]. The PSI network member in each country chose whether to use these tools, adapt them, or adopt other tools.

To further support the implementation of YFHS in private and public sector partner clinics, PSI provided YFHS training directly to franchise providers and staff, health officials, referral agents, and local youth, with the aim of expanding youth access to healthcare in facilities, i.e., by “mainstreaming” YFHS [27]. (Note that PSI does not operate clinics but, instead, engages independent private and public-sector health clinics in social franchise relationships to form healthcare networks.) An adaptable 3–5-day curriculum was used to educate participants on the unique FP/RH service needs of young people (10–24), youth (15–24), and adolescents (10–19) [27]. Curriculum topics included an overview of core YFHS components, discussion of adolescent development, and updates on contraceptive technology. Activities included examining the impact of provider and clinic staff’s personal values on youth access to and use of health resources and developing practical skills for communicating with and counseling youth in RH and reproductive rights [27]. Each training was tailored to fit the local country context and the needs of providers and staff. To help ensure that training was put into practice, PSI network member staff provided support such as on-site visits and on-going supervision to assist providers and staff in implementing YFHS in their clinics. At the global level, PSI also added a quality assurance standard related to the inclusion of young people, youth, and adolescents for all voluntary FP service delivery programs, regardless of funding source. With SIFPO2 support, PSI made an updated YFHS training curriculum available in 2015 [28].

The PSI program to mainstream YFHS implemented key elements of the High Impact Practices Framework for Providing Adolescent Friendly Contraceptive Services [12]. Key service delivery elements to overcome barriers were training and supporting providers to offer nonjudgmental services to adolescents, enforcing confidentiality and ensuring audio and visual privacy, offering a wide range of contraceptive methods, and providing free or subsidized services. Ensuring legal rights, policies, and guidelines to protect adolescent’s rights and fostering support among communities through outreach were key to building an enabling environment.

### Country context and program

Malawi has a young population, with two-thirds of the country’s population under the age of 25 [29]. Early marriage and young childbearing are common: among 20–24 year-old women, 42% were married by age 18, while 29% of 15–19 year olds have begun childbearing [29, 30]. The median age at first sex for women is 16.8 years, though only 15% of adolescent girls age 15–19 use a modern method of contraception [29]. The public sector is the most common source of FP services in the country; 79% of current female contraceptive users received their most recent supply from a public source [29]. This trend appears to be true for the youngest users as well: previous research found that 57% of female adolescents and 47% of male adolescents in Malawi preferred seeking RH care from public clinics over private or “other” sources, due to positive perceptions of confidentiality, accessibility and cost [31].

In 2012, PSI Malawi launched a social franchise network for health services, the Tunza Family Heath Network, to increase access to quality health services in Malawi. The Tunza Family Health Network has 69 private franchised clinics; PSI Malawi provides franchisees with training and mentorship in business management, quality assurance of clinical services, and YFHS. In 2017, 34% of Tunza Family Health Network FP clients were youth under 25 years of age.

PSI Malawi conducted YFHS training in 2013, 2014, 2016 and 2017 for 46 providers, 40 of whom remain active in the network. Trainings in 2016 and 2017 used the updated YFHS training curriculum, and were given as a refresher for some providers, and as a first training for newer franchise members. The curriculum was implemented within a context of additional and ongoing programs for youth, including community outreach and demand generation. The trained providers and staff offered a wide range of FP methods, including implants and copper intrauterine devices (IUDs) as long-acting, reversible contraception (LARC). “Youth Friendly” clinic branding certification was available.

### Evaluation purpose and objectives

The purpose of the evaluation was to contribute evidence on the effects of mainstreaming elements of adolescent- and youth-friendly health services into existing FP service delivery. More precisely, the evaluation assessed the changes in youth’s voluntary uptake of FP methods and perceptions of service quality. We hypothesized that YFHS training packages would improve the quality of mainstreamed services for youth, which in turn, would increase the number of youth accessing services and choosing voluntary FP methods. The evaluation also sought to assess the motivations of healthcare staff and their attitudes and behaviors towards youth as a result of the YFHS training packages. We hypothesized that the trainings and support from PSI Malawi would lead to provider and staff uptake of youth-friendly behavior, which in turn, would contribute to a perception of high service quality among youth.

## Methods

### Evaluation design

The evaluation used a mixed-methods convergent parallel design. The quantitative phase was a non-experimental, retrospective design which included desk review of information from relevant monitoring and evaluation documents triangulated with service statistics collected through PSI’s health information system. The qualitative phase incorporated data collected from focus groups and key informant interviews. Each phase was analyzed separately, and findings were integrated to generate conclusions and suggestions to improve and sustain quality YFHS. Research and data collection were led by the United States Agency for International Development-funded MEASURE Evaluation project at the Carolina Population Center, University of North Carolina at Chapel Hill. The data used for the evaluation are summarized below.

#### Program documents

Materials related to PSI activities to support the mainstreaming of YFHS in Malawi were collected and reviewed between June–October 2018. The documents included strategic documents, program reports and technical materials, such as technical briefs, supportive supervision checklists, and the YFHS training curriculum, facilitator’s guide and training workshop materials. Information requests were sent to points of contact at PSI Malawi and PSI Washington to clarify emergent questions.

#### Service statistics

The data available for the evaluation included monthly service data from January 2013–July 2018 from clinics in which at least one provider received YFHS training in 2013, 2014, 2016, or 2017. The data included number of services by age group (15–19 and 20–24) and FP method related to service (IUD, implant, oral contraceptive pill, injectable, condoms, or counseling only).

#### Qualitative data

Nine focus group discussions (FGD) were conducted, three with males ages 18–25 and six with females (two for ages 15–19 and four for ages 20–24) living in communities served by Tunza Family Health Network clinics that had worked to mainstream YFHS. The FGDs took place in the towns of Dowa, Kasungu, Mzuzu, Ekwendeni, Lilongwe, Nkhata Bay, and Nkhotakota in the central and northern regions of Malawi. Groups ranged in size from 4 to 13 youths—the group of four was a result of heavy rains that kept some recruited individuals from attending the discussion session. Following a convenience sampling approach, local organizations were contacted to assist with recruiting and finding space for the FGDs. Recruitment and discussions took place near the health facility or in program space in the selected community. Inclusion criteria for participants in FGDs (1) were youth ages 15–24, regardless of parity or marital status, and (2) had knowledge of the PSI network member healthcare facility, regardless of whether they had personally been a client. There were no exclusions based on gender, marital status, race, or ethnicity. Focus group participants were asked about their attitudes toward services offered to youth, perceptions of service quality at the facility, and whether the healthcare facility was seen as meeting the needs of youth in their communities. Focus group discussions lasted approximately 1 hour.

Key informant interviews were conducted with ten healthcare providers and staff that received the YFHS training since 2014. The sample allowed for about one-quarter of the overall number of trained health staff to provide input to the study. The ten healthcare providers, one per clinic, were purposively sampled among Tunza clinics to include different geographic locations and length of time since receiving the training. Staff eligible for interview were those who (1) received the training and materials for mainstreaming YFHS in the past 5 years, (2) were currently working in Tunza YFHS clinics, and (3) were available for the interview on the day of data collection. Staff eligible for the interviews were identified by the research team utilizing a list of all eligible clinics. An attempt was made to include a mix of service provider types (in-charges, physicians, counselors, and nurses). Service providers were not excluded by whether they currently serve adolescents and youth. Initial contact with selected staff was made by telephone; all who were contacted agreed to be interviewed. The communities in which the health staff interviews took place included Dowa, Kasungu, Mzuzu, Lilongwe, Nkhata Bay, and Nkhotakota. Healthcare providers were interviewed in a private space in their clinic and were asked about their attitudes on mainstreaming YFHS; their perceptions of successes and challenges to these efforts; attitudes on YFHS training; and perceptions on sustainability of the YFHS efforts. The interviews were structured around the WHO Quality of Care Framework standards for YFHS and the WHO Quality Assessment Guidebook for assessing health services for youth [20, 32]. Themes included access, acceptability, confidentiality, equity, and effectiveness [20]. Providers were also asked their opinions for improving YFHS efforts and areas for future work. Interviews lasted approximately thirty minutes.

The qualitative data were collected from November 25 to December 5, 2018, by Dr. Thakwalakwa (PhD) and Mr. Alfonso (MA), both with extensive experience in conducting key informant interviews and FGDs and fluent speakers of languages used in data collection. The FGDs were conducted in Chichewa. The key informant interviews were conducted in English. Interviewer guides included prompts when necessary and were reviewed by technical advisors at PSI. The FGDs and interviews were audio recorded, transcribed, and translated into English as needed.

### Analysis

Evaluation of PSI Malawi’s YFHS training and intervention package included a document review and identification and contextualization of information contained within program reports and strategy documents. Service statistics were provided in Excel. Descriptive statistics were then used to assess trends in FP services to youth. Graphs were developed to display trends. Client numbers were assessed by quarter, in an effort to smooth out data issues, such as the effects of reporting gaps for any single month. Content analysis of qualitative data according to themes used in the interview and focus group guides was undertaken to assess youth’s perception of service quality and healthcare staff’s perceptions of the YFHS training and implementation in Malawi. The analysis of qualitative data involved three iterative steps: reading, organizing and displaying, and reducing. First, a member of the study team read each transcript at least twice and highlighted sections of the transcripts to help bookmark quotes that were potentially meaningful or unexpected. Next, to organize and display the data, a matrix was developed in Excel to summarize typical and atypical responses to interview questions. The final step involved summarizing the findings and analyzing by respondent sex and age to identify relevant themes and patterns of responses. The final step was iterative and involved the entire research team. Information from documents, key informant interviews, and FGDs was used to contextualize the available service data and to identify and assess the strengths of implementation of a context-specific, multipronged intervention that paired YFHS provider and staff training with community outreach and demand-generation strategies. The analysis also identified barriers to effectiveness of YFHS provider and staff training, long-term perceptions of the YFHS training, and youth perceptions of barriers to accessing FP within the Malawian context and assessed whether the training was successful in increasing use of FP services among youth.

### Ethical considerations

In preparation for the activity, a memorandum of understanding for the sharing of data between PSI and MEASURE Evaluation was signed on November 10, 2017. The University of North Carolina Institutional Review Board approved the evaluation protocol and data collection tools, including consent forms, on August 24, 2018, through expedited review #18–1303. The Malawi National Committee on Research in the Social Sciences and Humanities approved the collection of qualitative data on November 12, 2018, through permit #P.09/18/318. Informed written consent and assent was obtained from all key informants and FGD participants. Providers and staff approached for participation in the study were informed that the interview was not required by Tunza nor would it influence their relationship or affiliation with PSI. A waiver of parental permission was received for participants aged 15–17, in accordance to section 4.1.2 of the Malawian National Commission for Science and Technology research framework, which states that parental permission may be waived for research involving adolescents about contraceptive access [33].

The anticipated risks of participating in FGDs included possible disclosure of personal information and the potential for feeling uncomfortable discussing RH topics. To reduce these risks, researchers emphasized that participants should not disclose personal information about their sexual behaviors, that what was discussed in the group should be kept confidential, and that participation in the discussion was voluntary and participants were free to refuse to answer any question or to leave at any time.

## Results

### Content of YFHS training program

The review of documents shows that in addition to the YFHS training offered in 2013, 2014, 2016, and 2017, supportive supervision by PSI Malawi reinforced the youth-friendly adaptations to clinics, as did the requirements for clinics to receive a “Youth-Friendly Clinic” certification (and branding), which almost all clinics completed within a year following YFHS training. PSI-trained interpersonal communication agents also engaged in one-to-one sessions with youth and referred them to Tunza health clinics. Partners conducted activities to increase community awareness and approval of youth use of FP services by engaging local community and religious leaders, presenting radio and television broadcasts, and conducting a broad range of communication and sensitization events. However, youth outreach programs concluded at the end of 2016. The number of youth clubs also declined in 2017.

### Service provision to youth

**Fig. 1.**
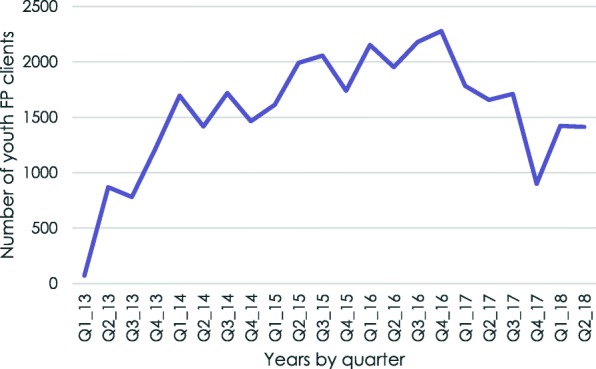
Total number of youth FP clients in YFHS training intervention clinics, Malawi

The five-year trend in the number of FP clients ages 15–24 seen at the 39 Tunza clinics in which staff received training is shown in Fig. 1. There is an increasing trend in numbers through most of the period with a peak of 2278 youth clients during the fourth quarter of 2016, after which the numbers begin to decline.

**Fig. 2.**
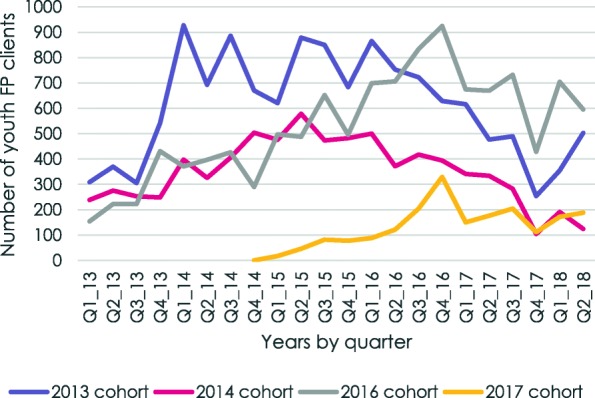
Number of youth FP clients in YFHS training intervention clinics, by training cohort, Malawi

Disaggregation of the clinic data by training cohort shows noticeable increases in the total number of FP clients in the months after the training. The initial increases, however, were not sustained. Notably, the 2013 and 2014 training cohorts were already showing declines in numbers by the time the 2016 and 2017 cohorts were reaching their peaks (Fig. 2).

**Fig. 3.**
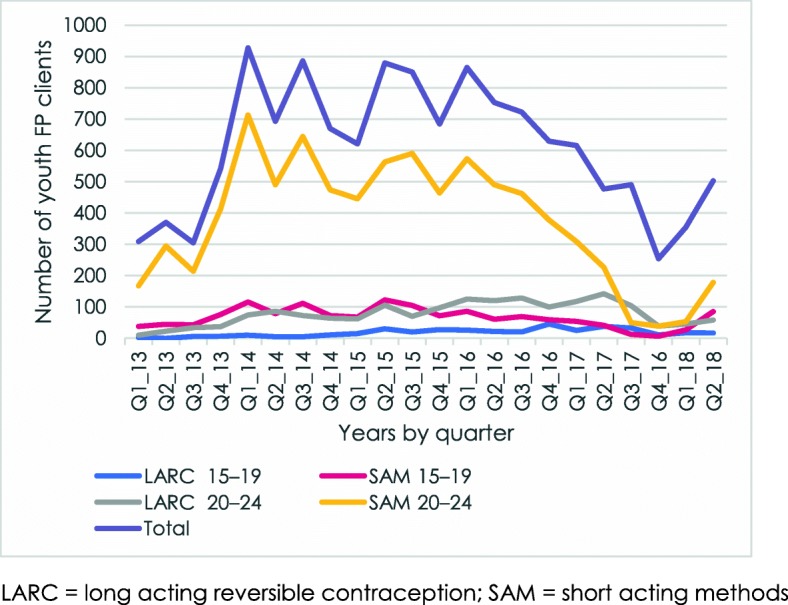
Number of youth FP clients served by 2013 YFHS training cohort, by age group and method choice, Malawi

The general pattern is replicated when looking at method choice (long acting and short acting) and age group (15–19 and 20–24), as shown for the 2013 training cohort in Fig. 3. The initial increase and subsequent decrease in numbers was driven mainly by clients ages 20–24 who chose short acting methods (SAMs) (specifically, the injectable). This pattern holds true for the other training cohorts as well, with only one exception: the clinics involved in the 2017 cohort show higher initial use of LARCs among the 20–24 age group (see Additional file 1: Figure S1-S3).

### Youth perceptions of service quality

In total, 49 females and 21 males participated in the nine FGDs. The distribution of groups by age and sex is shown in Table 1. The youth discussed issues related to the accessibility and acceptability of FP services at Tunza clinics. Some youth reported on their direct experiences, and others reported on what they had heard from their peers. Youth also provided suggestions for improving youth-friendly FP services. In general, there was little variation in responses provided by males and females, or by age group.

**Table 1:** Distribution of focus group discussion participants by age and sex

Focus groups	Number of FGDs	Number of participants	Average age of participants
Males 18–25	3	21	20.7
Females 15–19	2	14	16.7
Females 20–24	4	35	21.9
Total	9	70	20.5

#### Treatment of youth by staff

Youth involved in the FGDs were aware of a wide range of sources where they could access FP services, including at private clinics, such as Tunza clinics, public health facilities, pharmacies, Banja la Mtsogolo nonprofit health centers, youth clubs (mainly for condoms), and the Family Planning Association of Malawi, a nongovernmental organization. Youth were asked specifically about the treatment provided by their local Tunza Family Health Network private clinic. Among youth who had obtained FP services from a Tunza clinic, most reported that staff were “friendly,” “warm,” “approachable,” “respectful of privacy and confidentiality,” and “understanding” when people have money problems and that the staff provided information about different FP methods. One youth also appreciated the convenient location of the clinic and the speed of service compared to public health facilities.

*The doctor here always urges us to approach him for service, even if it means calling him. . . . He is always ready to assist us. He is always there for us.* —Male youth, 20–24

*People are warm. They teach us not to be shy around them.* —Female youth, 15–19

A participant in one of the female focus groups in the 20–24 age range added that the Tunza staff treat people well, better than at the government health facilities, “and faster too,” but it is because they are paying clients. In fact, long wait-times at a Tunza clinic was only mentioned in one focus group.

The youth were asked to comment on whether they thought FP services at the Tunza clinics would be private (not seen or overheard by others) and confidential (the clinic staff would not talk about their concerns with other clinic staff, clients, or parents). In most cases, both female and male youth reported that privacy and confidentiality at Tunza clinics was good:

*We feel there is confidentiality because the service providers are trained and they know their ethics. When we come here, they help us and do not tell anyone.* —Male youth, 20–24

One of the female groups (ages 20–24) added that even though there is confidentiality at the clinic, they are “just embarrassed to come.” However, in two of the male and one of the female groups near the seven clinic locations, youth were skeptical of confidentiality because the clinic staff were community members. Members of these groups stated they would prefer to obtain FP services from clinicians who were not from their community.

#### Barriers to accessing services

The youth did not perceive any barriers to FP services at Tunza clinics based on demographic characteristics. However, one of the male groups discussed that youth were lower in priority rankings relative to other age groups, perhaps because of the inability of some youth to pay. Overall, the youth reported that unmarried youth are able to access FP at Tunza clinics and that parental consent has never been required to their knowledge. Most of the youth groups also felt that even very young adolescent clients would be served by Tunza clinics. However, a member of one of the male discussion groups thought that the in-charge at the local Tunza clinic may want parental approval for a girl, because he might worry what would happen if she has complications. The group then clarified that parental consent has never actually been “required.” Participants in this male discussion group also thought the clinic in-charge would refuse services to someone he considered to be too young to be sexually active. (The in-charge was described as being “old.”) Belonging to a different ethnic group was not seen as a barrier to obtaining services at any of the Tunza clinics.

A main reason youth said that they or their peers did not access FP services at the Tunza clinic was because they had to pay a fee while they could access free FP services elsewhere (specifically, at a Banja la Mtsogolo health center or from the Family Planning Association of Malawi). While condoms are often free at Tunza clinics, most methods are not, and some methods, such as implants and IUDs, are expensive.

*We heard PSI subsidized family planning services at this clinic, but we wonder why the services are still on the higher side.* —Male youth, 20–24

*The prices are a challenge for the youth. Most of the youth wait for the adverts from Banja la Mtsogolo for free family planning services. So, in order not to spend money, they wait for Banja la Mtsogolo to advertise for the free services.* —Male youth, 20–24

Another common barrier to accessing FP services by both female and male youth was embarrassment. Feelings of embarrassment appeared to stem from internalized stigma associated with negative social norms about youth engaging in sexual activity. For example, some youth (both female and male) stated that they or their peers would be embarrassed if they were seen entering a Tunza clinic by a friend, because the friend might conclude they are there for FP, which is one of Tunza’s highly promoted services:

*Friends may see you and start asking questions like, “what is she doing there? If she is there then she has to be going there for the condoms, or is she pregnant.”* —Female youth, 20–24

*You may come to get condoms here, but before entering the gate, you start asking yourself questions, “what will I say if people ask me what I was doing here?” . . . Unlike other facilities, this one does not have bawo* [a board game] *or chess.* [that could be used as an explanation for being in the clinic] —Male youth, 20–24

The female groups discussed the clinics with multiple services, saying a person could pretend to go for treatment of malaria or because of other illness, to avoid anyone finding out the person wanted FP services. However, this tactic was reported as being difficult if the clinic was too small or if the visit was during specific FP hours. For example,

*At a Tunza clinic you have access to any birth control method you would like. However, the clinic offers family planning [to youth] on the same days as to older women, so it is not as private. —*Female youth, 20–24

Another female group added that women who have had children do not experience the same level of embarrassment.

As mentioned above in relation to treatment provided by the staff, Tunza clinic staff serving within their own communities was also seen as a potential barrier to accessing services. Youth in three FGDs reported they or their peers did not access services at the Tunza clinic because the clinic staff were community members, and this made the youth uncomfortable:

*The workers in this clinic live with us in the neighborhood and some of them are related to us. Some are friends with our parents … so we fear that they will end up telling them that we visit the clinic for family planning services.* —Male youth, 20–24

#### Suggestions for improvement

Finally, the youth were asked if they had any suggestions for improving FP services for young people at the Tunza clinic. The youth offered a variety of suggestions. A common response was the importance of FP services and methods being free of charge or offered at reduced prices to youth. Another common response was that youth would like to see youth clubs restored at clinics, with someone appointed to support or supervise the club and organize health talks. Other youth reported they would like the clinic to have a dedicated room for youth with games such as *bawo* and booklets about FP, or specific days set aside for youth to obtain FP services. One male group suggested more interaction between the in-charge and youth, stating that “youth and service providers can only be free with each other if they interact frequently.”

### Healthcare provider attitudes and behaviors

Ten healthcare staff (nine male and one female) from Tunza Family Health Network clinics participated in key informant interviews. Five of the staff were clinic in-charges, three were clinic directors, one was a nurse, and one was an HIV testing assistant. Three had received their most recent training from PSI in 2013, three in 2014, two in 2016, and one in 2017. One provider misreported being trained in 2015. Respondents had been in their current positions an average of 11 years, with a range of 1–27 years. Six of the clinics were located in more rural areas, and four were in urban areas.

Providers discussed accessibility of FP services for youth, acceptability of FP methods for youth, and the importance of confidentiality in providing services to youth. They also expressed their views on mainstreamed versus stand-alone youth-friendly clinics and provided suggestions for improving the Tunza Family Health Network initiative.

#### Accessibility

The interviewed healthcare staff were first asked to describe what it means to make FP services accessible to youth. The staff reported a variety of ways that FP services can be made accessible. These included providing services for free or at reduced cost, being friendly to youth and treating them with respect, educating youth about FP methods, and ensuring confidentiality. One of the providers summed the issues up in this way:

[To be accessible] *there should be provision of free services for youth, and also, there should be confidentiality in the environment in which these services are provided. In addition, the people who provide these services, are they youth-friendly? Some* [youth] *are afraid of meeting old personnel who they feel might judge them for their actions.* —Tunza service provider

Another provided the following description of accessibility:

*Spending time with youth and discussing health-related issues with them to prevent them from making bad decisions.* —Tunza service provider

One provider mentioned easy access to FP services, regardless of age, “assuming they are over 12.” The healthcare staff emphasized that provision of free or reduced price FP services is key to making the services accessible to youth:

*The most important thing is the services must be free. For example, when we are offering free services or subsidized services from PSI, we get a lot of young people coming in to access the services. This means a lot of the youth want these services and we can achieve much if we have free services.* —Tunza service provider

However, several healthcare staff reported that their clinic was only able to offer free or reduced services when supported by PSI.

*Interviewer: Are the young people required to pay for particular services?*

*Respondent: Oh yes. They pay for all the services.*

*Interviewer: Is there any difference in terms of the payments between the youth and adults?*

*Respondent: No, there is no difference. Only during certain periods we are told the youth should be treated for free, that they should be given free services. But mostly, they pay the whole amount and only when it is offered by PSI can we allow them to receive the services free of charge.* —Tunza service provider

To improve accessibility, build rapport with youth, and create an avenue for educating youth about FP and other health issues, some clinics have games available, such as bawo or chess, or have youth clubs for football or netball, with games and balls provided by PSI. To promote community awareness of FP services at their clinic, some healthcare staff reported that they gave health talks to youth clubs, have community outreach personnel that educate families about FP services and other health issues, and mobilize youth when they are offering free FP services on a particular day. In contrast, a few providers did not conduct outreach and felt that community awareness of services was low.

Overall, most providers thought the steps they had taken to make FP services accessible to youth were successful, citing increased numbers of youth clients as evidence; however, none had formally assessed youth satisfaction.

#### Acceptability

Most providers and staff reported that youth in their clinics were allowed to choose the FP method each found most acceptable after counseling was provided on all methods:

*Providing family planning methods that are acceptable by the youth entails providing information about all the methods and helping the youth make an informed decision on their preferred choice.* —Tunza service provider

However, a couple of providers stated they did not counsel youth on LARC, specifically implants and IUDs, stating that youth do not like those methods because they fear they will never have a child if they use them. Nearly all providers reported that condoms, oral contraceptive pills, injectables, and emergency contraceptive pills were the methods most requested by youth, noting that condoms are primarily accessed by males and that females generally access the other methods. Providers stated that these short-term methods are safe and appropriate for youth and reiterated that it is rare for youth to request LARCs.

#### Confidentiality

Providers unanimously stressed the importance of confidentiality in providing services to youth:

*Confidentiality is a primary thing. You have to make a person understand that whatever they are going to volunteer to you is going to be confidential, and it is emphasized that if a person wants a third party to be involved, that would be up to them.* —Tunza service provider

*I tell the youth, “whatever we discuss here will be between you and me. No one apart from us will know.”* —Tunza service provider

A few providers reported that they allowed youth to enter their clinic through a special door or provided a special room for youth consultations to help ensure privacy and confidentiality. Others employed young people at reception whom the youth could chat with while waiting for services—in this way, if youth were seen at the clinic, they could say they were just visiting a friend (the receptionist). A few reported that youth were not made to wait in a queue but were brought to see a provider immediately.

#### Equity

None of the providers stated that they would require parental consent to provide FP services to youth under age 17. However, a few reported that they were “uncomfortable” or “reluctant” to provide services to youth under age 15; in one of the cases the provider stated he would be hesitant to provide services to a youth under age 13. With regard to providing FP services to married females under 17, only one of the providers reported that he would require the husband’s involvement. Other providers stated that involving the husband would be the choice of the woman:

*She is the one who has the choice. It is not for the husband. . . . If she wants the husband to be there, she is free to do so. The choice is hers.* —Tunza service provider

*Informing the husband is the responsibility of the woman*. —Tunza service provider

With regard to other groups that providers do not feel comfortable serving, a few providers mentioned “drunk youth” and one mentioned “Jehovah’s Witnesses” (because “they do not like FP”).

#### Effectiveness: mainstreamed compared to stand-alone youth-friendly clinics

The healthcare providers and staff had mixed views of whether mainstreamed youth-friendly clinics or stand-alone youth-friendly clinics were more accessible for youth. Their views were based on the issue of which type of clinic could potentially offer the most confidentiality. Some healthcare staff felt that youth could access mainstreamed clinics with greater confidentiality because they could be visiting such clinics for a variety of reasons: FP, malaria treatment, or others. On the other hand, some staff felt that stand-alone clinics offered more confidentiality because youth would not have to worry about seeing older family or community members there.

#### Experience with PSI and suggestions for improvement

The interviewed providers gave positive feedback regarding the training they received from PSI, noting they learned how to approach youth and understand their needs, challenges, and fears with regards to FP. After the training, providers reported receiving items for youth such as footballs, netballs, bawo, and booklets. However, many providers reported that follow-up by PSI after the training was infrequent, with a few reporting that promised materials (such as condoms) were not delivered or that promised activities (such as youth club trips) never materialized. These providers felt this was discouraging to youth:

*They* [PSI] *should be fulfilling the promises they make to the youth, otherwise they are demotivated and stop participating in youth activities. For instance, the time they* [the youth] *were promised a trip to Salima, membership of our club rose, but the number has dropped because there is nothing happening on the ground.* —Tunza service provider

This also made it difficult to continue with some of the activities seen as helpful in improving accessibility.

*Interviewer: Were there any changes that you made initially, that you do not continue? If so, what were they and why were they not continued?*

*Respondent: Yes, the youth club. We have it, but it is not very active because when we started the club, we drafted a proposal, and after submitting it, they* [PSI] *promised to provide us with materials like flip charts and other materials to use at the club. However, it just ended there. The only thing we received was a football. So this just frustrated the club members, thinking that we were perhaps benefiting from the materials ourselves.*

*Interviewer: Is there any other change that you made initially that did not continue?*

*Respondent: We also introduced youth talks within the surrounding schools. We would go and have talks with the youth and also invite role models to go and talk to the students. But that also did not continue.*

*Interviewer: What caused this not to continue?*

*Respondent: The problem was that there was lack of motivation from the people assisting us, since there were no incentives. There was no good coordination with our partners, hence it was like a ‘one man show’.* —Tunza service provider

The healthcare staff offered suggestions for strengthening the Tunza Family Health Network youth-friendly initiative, which included adding a hands-on practical component to the initial training, adding refresher trainings, convening meetings of providers so they can share experiences and learn from each other, providing on-going guidance and feedback on their practices, and training youth as peer educators or youth coordinators to manage and organize youth clubs and activities.

## Discussion

Evidence suggests that the most effective programs to improve youth access to and uptake of FP/RH services do not provide YFHS training for clinic staff and providers as an isolated intervention; rather, they package interventions addressing multiple components of providing successful YFHS. Denno, Hoopes, and Chandra-Mouli (2015) found that programs are more successful when interventions combine (1) YFHS training and (2) adaptation of facilities with at least one other core component, such as (3) demand generation for services and/or (4) broad information dissemination campaigns [8]. In alignment with this evidence, the evaluation of efforts to mainstreaming YFHS in the Tunza Family Health Network found the delivery of the YFHS training curriculum was successfully packaged with multiple components. However, despite the training and adaptions made to the facilities, declines in youth client numbers occurred after YFHS training and youth outreach programs were discontinued in 2016 and 2017. These results are similar to an evaluation of mainstreaming YFHS efforts by PSI networks in Madagascar and Mali [34]. For example, results from the Madagascar franchise show substantial declines in the number of youth clients after funding, vouchers, and peer education activities ended. The assessment of client numbers by training cohort in Malawi also suggests that motivation of providers may play a factor, with motivation to serve youth high immediately after a YFHS training and declining over time. The results suggest that provider training and facility adaptations are not sufficient to sustain initial positive increases in youth client numbers without continuing efforts in demand generation and informational campaigns.

Findings from the FGDs indicate that, overall, certified Tunza clinics in which at least one provider had undergone YFHS training between 2013 and 2017 were still perceived as providing high-quality services to youth. Youth FGD participants commonly used positive words, such as “respectful” and “friendly” to describe services. There were no known barriers to service provision based on demographic barriers. Importantly, most youth thought privacy and confidentiality were protected at the clinics. However, the issue continued to be problematic for youth living in small communities in which the provider(s) may know the youth, their family, or their friends. These youth would be hesitant to go to the clinics despite the overall positive assessment of services.

Cost remains a significant barrier to FP/RH services for youth. While the issue is recognized by Tunza program staff and service providers, it appears the franchise clinics do not have a clear path to providing free or subsidized services to youth without financial support from PSI. Programs should continue to explore potential solutions, including domestic financing, for long-term provision of FP/RH services for youth. For their part, the youth seemed aware of places to receive free FP methods and to know that free services are not always consistently available, depending on the provider. Inconsistent access to services or contraceptive methods could lead to disruptions in care and use, with potentially negative health outcomes.

Finally, some providers feel that interest in LARC is low among youth and, as a result, do not always offer these methods as options. Such practices could reinforce negative attitudes of youth toward LARC and undermine uptake of these methods. In accordance with the global consensus statement on expanding youth access to contraceptives to include LARCs, pre-service and in-service mentoring and coaching with providers and demand-generation efforts with young people can help reinforce that LARCs are appropriate methods for young people [35].

### Strengths and limitations

Strengths of the evaluation included the use of established YFHS frameworks to guide the study design, data collection, and analysis. The study design and implementation also benefitted from the participation and support of stakeholders, including local PSI Malawi and Tunza clinic staff. The collection of qualitative data allowed for the assessment of important outcomes of provider training that were not tracked through service statistics.

The retrospective design of the evaluation meant that analysis of program and service data was constrained by the information collected by the program. Additionally, due to the non-experimental design, secular increases or decreases in the trend of FP use or other non-programmatic factors could influence the interpretation of results.

Qualitative data provided important information on perceptions of YFHS programming and quality of care. However, the recruitment of FGD participants ages 15–17 was difficult because it occurred during end-of-semester testing, and youth that were still in school were either busy with test preparation or too tired to participate after the school day. As a result, few youth ages 15–17 were recruited and we were not able to make comparisons between the youngest and oldest youth. The sample is also missing the voices of the youngest men, ages 15–17.

Finally, additional research is needed to assess the sustainability of short-term outcomes, including increases in the number of youth FP clients. This evaluation provides evidence that short-term improvements in the number of youth clients are not sustained when essential elements of YFHS interventions are discontinued; however, more information is needed to determine the amount of programmatic effort needed to sustain initial improvements.

## Conclusions

The findings support research showing positive effects of mainstreaming YFHS when training for healthcare staff combined with changes to make facilities more youth friendly is implemented with demand-generation activities, such as outreach through youth groups and clubs; increased presence at community events; and radio, television, or other mass media approaches. However, the sustainability of YFHS intervention packages is an issue that needs attention. The results show that without sustained outreach and demand-generation activities and the provision of free or reduced-cost RH services to those with a financial need, initial increases in youth clients will not be sustained. Based on this evidence, to improve young peoples’ health and access to FP resources, provision of YFHS should include effective training of providers and staff while also considering the ongoing structural, financial, and community contexts in which youth FP and RH services are provided.

## Supplementary information

**Additional file 1: Figure S1-S3.** Number of youth FP clients served by 2014, 2016, 2017 YFHS training cohort, by age group and method choice, Malawi.

## Data Availability

Programmatic information on PSI efforts to mainstream YFHS, including training curriculum, guides, and reports, are available through the PSI website: https://www.psi.org. Service statistics used for the analysis may be made available upon reasonable request to co-author Ashley Jackson. Qualitative data are not publicly available due to potential disclosure of information that could compromise research participant privacy.

## Abbreviations

FGD: Focus group discussion
FP: Family planning
IUD: Intra-uterine device
LARC: Long-acting reversible contraception
PSI: Population Services International
RH: Reproductive health
SAM: Short-acting method
SIFPO: Support for International Family Planning Organizations
YFHS: Youth-friendly health services
