# Supplementary material for: Evaluation of mainstreaming youth-friendly health in private clinics in Malawi
Source: BMC Health Serv Res. 2020 Feb 3;20:79. doi: 10.1186/s12913-020-4937-9 (PMC6998314; doi:10.1186/s12913-020-4937-9)
Supplement: Supplementary file 1 — Additional file 1: Figure S1-S3. Number of youth FP clients served by 2014, 2016, 2017 YFHS training cohort, by age group and method choice, Malawi. [file 12913_2020_4937_MOESM1_ESM.docx]

**Figure S1. Number of youth FP clients served by 2014 YFHS training cohort, by age group and method choice, Malawi**

**Figure S2. Number of youth FP clients served by 2016 YFHS training cohort, by age group and method choice, Malawi**

**Figure S3. Number of youth FP clients served by 2017 YFHS training cohort, by age group and method choice, Malawi**
